# Supplementary material for: Content-rich biological network constructed by mining PubMed abstracts
Source: BMC Bioinformatics. 2004 Oct 8;5:147. doi: 10.1186/1471-2105-5-147 (PMC528731; doi:10.1186/1471-2105-5-147)
Supplement: Additional File 5 — The original Chilibot query results of the term "long-term potentiation (LTP)" and 22 other terms, limiting the latest references analyzed to the years 1990, 1995, 2000, and 2004. [file 1471-2105-5-147-S5.bz2 › chilibotAdditionalFile5/ltp2004/html/AMPA.html]

 


**AMPA** (Input: AMPA ) 

---


**Google Searches:** Entire Web  | EDU domain only  | PDF files only

.

**External Links:** OMIM |
LocusLink
| Swissprot
| GeneCards

  
**Maps of AMPA**

Simple
Complete
graph in
radiant
tree
square
layout.

**New Hypothesis !**

**Synonyms** 

- ampa   [PubMed]

**Synopsis**

- Our results show that in the developing hippocampus synaptic plasticity can be expressed by **AMPA** receptors lacking the GluR A subunit.  J PhysiolJ Physiol, 2003 
  [25]

- These results suggest that common mechanisms are involved in controlling synaptic **AMPA** receptors by PSD 95 and synaptic plasticity.  J Neurosci, 2003 
  [25]

- These results suggest that 1 modulation of **AMPA** receptors which increases either response amplitude or duration can facilitate LTP formation, 2 modulators that effectively slow response deactivation augment the maximum magnitude of LTP and LTD, and 3 receptor desensitization may have a minor impact on synaptic plasticity in the hippocampus.Taken together, our data indicate that **AMPA** receptor modulators differ substantially in their ability to enhance synaptic potentiation or depression, depending on their particular influence on receptor kinetics, and hence that they may be differentially effective in influencing higher order processes such as memory encoding.  Neuroscience, 2004 
  [22]

- Together the data suggest that the highly regulated movement of GluR1 containing **AMPA** receptors between extrasynaptic and synaptic receptor pools is critically involved in establishing two functionally diverse forms of synaptic plasticity LTP and distance dependent scaling.  J Physiol, 2003 
  [22]

- These results suggest that, in hippocampal cultures, bFGF modulates **AMPA** and metabotropic glutamate receptors linked to the IP cascade, possibly in relation to the regulation of neuronal survival and glial cell proliferation, respectively.  Eur J Neurosci, 1999 
  [20]

- A widely accepted mechanism that has been suggested for synaptic silence is that functional **AMPA** glutamate receptors AMPARs are absent on the subsynaptic membrane.  Neuropharmacology, 2003 
  [19]

- These results suggest that GluR2 containing **AMPA** receptors, possibly within the CeA, are critical for the formation of stimulus reward associations necessary for PIT and conditioned approach, but are NOT involved in the plastic processes underlying the attribution of motivational value to the conditioned stimulus CS .  J Neurosci, 2003 
  [19]

- Because we have previously shown that acute ethanol can inhibit NMDA and **AMPA**,   Alcohol Clin Exp Res, 1999 
  [19]

- This reduction was in part caused by an apparently twofold reduction in mean quantal amplitude, as shown by recording **AMPA** receptor mediated miniature PSCs.  J Neurosci, 1999 
  [18]

- Here, we show that epileptiform activity strengthens excitatory hippocampal synapses by increasing the number of functional RS alpha amino 3hydroxy 5methyl 4 isoxadepropionate **AMPA** type glutamate receptors in CA3 CA1 synapses.  J Physiol, 2004 
  [16]

- Here we show that overexpression of the glutamate receptor 2 GluR2 subunit of **AMPA** receptors increases spine size and density in hippocampal neurons, and more remarkably, induces spine formation in GABA releasing interneurons that normally lack spines.  Nature, 2003 
  [15]

- However, we cannot exclude a role of PSD 95 in early events that lead to clustering of NMDA receptors or of other proteins including stargazin and **AMPA** receptors at postsynaptic sites nor do these experiments address the possibility of long term changes in the slices.  Neuropharmacology, 2003 
  [15]

- However, these mutant mice are competent in establishing several forms of long lasting synaptic changes in the CA1 region of the hippocampus, including LTD, long term potentiation  [LTP]  LTP, depotentiation, and dedepression, indicating the presence of GluR2 3 independent mechanisms of LTD expression and suggesting that **AMPA** receptor GluR1 alone is capable of various forms of synaptic plasticity.  Neuron, 2003 
  [13]

- Here we demonstrate the role of calcium calmodulin activated type I adenylyl cyclase AC1 in regulating the strength of thalamocortical synapses through modulation of **AMPA** receptor AMPAR trafficking using barrelless mice, a mutant without AC1 activity or cortical barrel maps.  Nat Neurosci, 2003 
  [13]

- In both systems, synaptic **AMPA** receptors can be defined based on colocalization of GluR1 and the synaptic marker synaptobrevin.  Ann N Y Acad Sci, 2003 
  [12]
